# Calculation of statistic estimates of kinetic parameters from substrate uncompetitive inhibition equation using the median method

**DOI:** 10.1016/j.dib.2017.03.013

**Published:** 2017-03-11

**Authors:** Pedro L. Valencia, Carolina Astudillo-Castro, Diego Gajardo, Sebastián Flores

**Affiliations:** a Department of Chemical and Environmental Engineering, Universidad Técnica Federico Santa María, PO Box 110-V, Valparaíso, Chile; b Escuela de Alimentos, Pontificia Universidad Católica de Valparaíso, Valparaíso, Chile; c Department of Mathematics, Universidad Técnica Federico Santa María, Valparaíso, Chile

**Keywords:** Direct linear plot, Median method, Substrate inhibition, Kinetic constants estimation

## Abstract

We provide initial rate data from enzymatic reaction experiments and tis processing to estimate the kinetic parameters from the substrate uncompetitive inhibition equation using the median method published by Eisenthal and Cornish-Bowden (Cornish-Bowden and Eisenthal, 1974; Eisenthal and Cornish-Bowden, 1974). The method was denominated the direct linear plot and consists in the calculation of the median from a dataset of kinetic parameters *V_max_* and *K_m_* from the Michaelis–Menten equation. In this opportunity we present the procedure to applicate the direct linear plot to the substrate uncompetitive inhibition equation; a three-parameter equation. The median method is characterized for its robustness and its insensibility to outlier. The calculations are presented in an Excel datasheet and a computational algorithm was developed in the free software Python. The kinetic parameters of the substrate uncompetitive inhibition equation *V_max_*, *K_m_* and *K_s_* were calculated using three experimental points from the dataset formed by 13 experimental points. All the 286 combinations were calculated. The dataset of kinetic parameters resulting from this combinatorial was used to calculate the median which corresponds to the statistic estimator of the real kinetic parameters. A comparative statistical analyses between the median method and the least squares was published in Valencia et al. [3].

**Specifications Table**

**Table:** 

Subject area	*Biochemistry*
More specific subject area	*Enzyme kinetics*
Type of data	*Tables, text file, graph, figure*
How data was acquired	*Simulated data of initial reaction rate*
Data format	*Raw and analyzed output data*
Experimental factors	
Experimental features	*Initial reaction rates were generated using the substrate uncompetitive inhibition equation with real values V*_*max*_*= 1, K*_*m*_*= 1 and K*_*s*_*= 100 and relative error from a normal distribution with standard deviation of 0.5*
Data source location	
Data accessibility	*Data is with this article*

**Value of the data**

• The data and calculations involved in the application of the direct linear plot to a three-parameter equation were described.
• The data arisen from this application was explicitly exposed and procedures explained.
• The data allows to visualize the advantages of the direct linear plot when applied to complex equations.
• Datasheets and algorithms can be used to generate new data and analysis to compare the direct linear plot with other estimation methods.

## 1 Data description

The raw data consists in initial rates from enzymatic reaction considering the substrate uncompetitive inhibition equation. This data was generated through simulation of the initial rate calculated from the substrate uncompetitive inhibition equation adding a relative error from a normal distribution with standard deviation 0.5. The analyzed data was a list of kinetic parameters *V_max_*, *K_m_* and *K_s_* obtained using the direct linear plot method [1], [2]. The resulting data was the statistic estimators of *V_max_*, *K_m_* and *K_s_* calculated from the median of the previous list.

## 2 Experimental design and methods

### 2.1 Calculation of initial rates

The dataset of initial reaction rates was obtained calculating *v*_*i*_ from Eq. (1) using the substrate concentrations displayed in Table 1.

(1) $$v_{i}=\frac{V_{\max}S_{i}}{K_{m}+S_{i}+\frac{S_{i}^{2}}{K_{S}}}\left(1+\varepsilon_{i}\right)$$

**Fig. 1:**
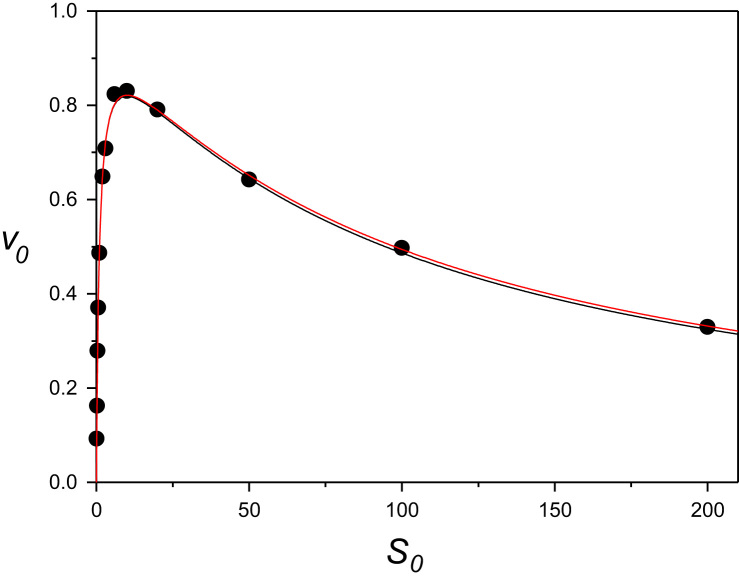
Initial rate versus substrate concentration dataset calculated from the substrate uncompetitive inhibition equation (points) and model curves with estimated kinetic constants from direct (black line) and inverse (red line) calculation of *K*_*s*_.

A normal error distribution was used to simulate and add the experimental error to each value of initial rate. The real values of kinetic constants were *V*_*max*_ = 1, *K*_*m*_ = 1 and *K*_*s*_ = 100. The standard deviation of the normal distribution of error was 0.5. The resulting dataset with the initial rate values is shown in Table 1 and plotted in Fig 1. It is important to notice that different datasets are obtained every time the calculations are done due to the aleatory condition of error.

### 2.2 Estimation of kinetic constants

The dataset in Table 1 was used to calculate the kinetic constants *V*_*max*_, *K*_*m*_ and *K*_*s*_ of Eq. (1) using the following equations for each constant.

(2) $$V_{\max}=\frac{v_{1}v_{2}v_{3}\left[\frac{S_{1}}{S_{2}}-\frac{S_{2}}{S_{1}}+\frac{S_{3}}{S_{1}}-\frac{S_{1}}{S_{3}}+\frac{S_{2}}{S_{3}}-\frac{S_{3}}{S_{2}}\right]}{v_{1}v_{2}\left[\frac{S_{1}}{S_{2}}-\frac{S_{2}}{S_{1}}\right]+v_{1}v_{3}\left[\frac{S_{3}}{S_{1}}-\frac{S_{1}}{S_{3}}\right]+v_{2}v_{3}\left[\frac{S_{2}}{S_{3}}-\frac{S_{3}}{S_{2}}\right]}$$

(3) $$K_{m}=\frac{v_{1}v_{2}\left(S_{2}-S_{1}\right)+v_{1}v_{3}\left(S_{1}-S_{3}\right)+v_{2}v_{3}\left(S_{3}-S_{2}\right)}{v_{1}v_{2}\left[\frac{S_{1}}{S_{2}}-\frac{S_{2}}{S_{1}}\right]+v_{1}v_{3}\left[\frac{S_{3}}{S_{1}}-\frac{S_{1}}{S_{3}}\right]+v_{2}v_{3}\left[\frac{S_{2}}{S_{3}}-\frac{S_{3}}{S_{2}}\right]}$$

(4) $$K_{S}=\frac{v_{1}v_{2}\left[\frac{S_{1}}{S_{2}}-\frac{S_{2}}{S_{1}}\right]+v_{1}v_{3}\left[\frac{S_{3}}{S_{1}}-\frac{S_{1}}{S_{3}}\right]+v_{2}v_{3}\left[\frac{S_{2}}{S_{3}}-\frac{S_{3}}{S_{2}}\right]}{v_{1}v_{2}\left[\frac{1}{S_{1}}-\frac{1}{S_{2}}\right]+v_{1}v_{3}\left[\frac{1}{S_{3}}-\frac{1}{S_{1}}\right]+v_{2}v_{3}\left[\frac{1}{S_{2}}-\frac{1}{S_{3}}\right]}$$

A data list consisting of 286 values for each kinetic constant was obtained from Eqs. (2), (3), (4). In the case of *K_s_*, the calculation can be made from Eq. (4) or from the inverse of Eq. (4). The difference between both methods is explained in the article Valencia et al. [3]. An incomplete list of results is shown in Table 2. The complete dataset can be found in Supplementary material in the file Median method.xlsx.

The estimated parameters for the kinetic constants of the substrate uncompetitive inhibition equation were obtained from the median of each parameter. The median can be calculated automatically with the function *Median* in Excel. The median estimators of the kinetic constants are listed in Table 3 along with the estimators obtained from the least-squares method.

An algorithm was developed in the free software *Python* to calculate the median estimator of *V*_*max*_, *K*_*m*_ and *K*_*s*_ from a dataset of initial rate versus substrate concentration can be found in Supplementary material in the file python.rar.

## Figures and Tables

**Table 1:** Dataset of substrate concentrations and initial rates obtained from Eq. (1).

***n***	***S***_**0**_	***v***_***0***_
1	0.1	0.092
2	0.2	0.162
3	0.4	0.279
4	0.6	0.370
5	1.0	0.487
6	2.0	0.649
7	3.0	0.708
8	6.0	0.824
9	10	0.830
10	20	0.791
11	50	0.642
12	100	0.497
13	200	0.329

**Table 2:** Dataset (partial) of estimated kinetic constants *V_max_*, *K_m_* and *K_s_* calculated from Eqs. (2), (3), (4).

**n**	***S***_***1***_	***S***_***2***_	***S***_***3***_	***v***_***1***_	***v***_***2***_	***v***_***3***_	***V***_***max***_	***K***_***m***_	***K***_***s***_	**1/*****K***_***s***_
1	200	100	50	0.330	0.497	0.642	1.145	8.816	82.3	0.0121
2	200	100	20	0.330	0.497	0.791	1.043	2.071	92.9	0.0107
3	200	100	10	0.330	0.497	0.830	1.032	1.372	94.2	0.0106
⋮	⋮	⋮	⋮	⋮	⋮	⋮	⋮	⋮	⋮	⋮
284	0.600	0.400	0.200	0.370	0.279	0.163	0.896	0.909	−6.28	−0.159
285	0.600	0.400	0.100	0.370	0.279	0.092	0.720	0.684	−3.08	−0.324
286	0.400	0.200	0.100	0.279	0.163	0.092	0.517	0.468	−1.26	−0.796

**Table 3:** Statistic estimators of the kinetic constants of the substrate uncompetitive inhibition equation.

**Kinetic constant**	**Median estimator**	**Least-squares estimator**
*V*_*max*_	0.984	0.996
*K*_*m*_	1.000	1.028
*K*_*s*_	98.73	98.57
*K*_*s*_*from 1/ K*_*s*_	101.9	–
