# Supplementary material for: Calculation of statistic estimates of kinetic parameters from substrate uncompetitive inhibition equation using the median method
Source: Data Brief. 2017 Mar 11;11:567–71. doi: 10.1016/j.dib.2017.03.013 (PMC5357693; doi:10.1016/j.dib.2017.03.013)
Supplement: Supplementary file 1 — Supplementary material [file mmc1.pdf]

AUTHOR DECLARATION

We wish to confirm that there are no known conflicts of interest associated with this publication and there has been no significant financial support for this work that could have influenced its outcome.

We confirm that the manuscript has been read and approved by all named authors and that there are no other persons who satisfied the criteria for authorship but are not listed.

We further confirm that the order of authors listed in the manuscript has been approved by all of us.

We understand that the Corresponding Author is the sole contact for the Editorial process (including Editorial Manager and direct communications with the office). He is responsible for communicating with the other authors about progress, submissions of revisions and final approval of proofs. We confirm that we have provided a current, correct email address which is accessible by the Corresponding Author and which has been configured to accept email from pedro.valencia@usm.cl

Signed by Pedro L. Valencia on behalf of all authors.

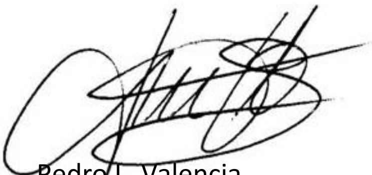

Pedro L. Valencia
Ph.D. Biochemical Engineering
Research Associate
Department of Chemical and Environmental Engineering
Universidad Técnica Federico Santa María

Phone: 56 322652718
Mobile: 56 9 92783228
Email: pedro.valencia@usm.cl
